# 

**Published:** 1908-06-27

**Authors:** 


					B11
GLT'-HAx:
^?W.o A,dr?.J JLl 7- li^HE MODERN
?. London. II MEDICAL JOURNALS
?Ef?lES* No- 68? Vo1
Stmo. Voi'xil,;?'- Satobday,
June 27th, 1908.
PRICE THREEPENCE.

				

